# The Sunshine Paradox: Unraveling Risk Factors for Low Vitamin D Status Among Non-Pregnant Women in Lebanon

**DOI:** 10.3390/nu17050804

**Published:** 2025-02-26

**Authors:** Carla El-Mallah, Amirhossein Yarparvar, Valeria Galetti, Omar Obeid, Mira Boutros, Gloria Safadi, Razan ZeinEddine, Nour El Hoda Ezzeddine, Maya Kouzeiha, Diana Kobayter, James P. Wirth, Mirella Abi Zeid Daou, Farah Asfahani, Nadeen Hilal, Randa Hamadeh, Firass Abiad, Nicolai Petry

**Affiliations:** 1 GroundWork, 7036 Fläsch, Switzerland; carla@groundworkhealth.org (C.E.-M.); valeria@groundworkhealth.org (V.G.); james@groundworkhealth.org (J.P.W.); 2 United Nations Children’s Fund, Beirut 1100, Lebanon; ayarparvar@unicef.org (A.Y.); mboutros@unicef.org (M.B.); gsafadi@unicef.org (G.S.); nezzeddine@unicef.org (N.E.H.E.); 3 Department of Nutrition and Food Sciences, Faculty of Agricultural Sciences, American University of Beirut, Beirut 1107, Lebanon; oo01@aub.edu.lb (O.O.); rz50@aub.edu.lb (R.Z.); 4 Mercy-USA for Aid and Development, Tripoli 1300, Lebanon; maya.k.kouzaiha@gmail.com (M.K.); diana.kobayter@gmail.com (D.K.); 5 World Food Programme, Beirut 1552, Lebanon; mirella.abizeid@wfp.org (M.A.Z.D.); 6 World Bank, Beirut 8577, Lebanon; fasfahani@worldbank.org (F.A.); 7 Ministry of Public Health, Beirut 1107, Lebanon; nadeenhilal@gmail.com (N.H.); randa_ham@hotmail.com (R.H.); firassabiad@gmail.com (F.A.)

**Keywords:** vitamin D, deficiency, hypovitaminosis, risk factors, hijab, sun exposure, supplementation, prevalence, reproductive-age women, Lebanon

## Abstract

**Background/Objectives**: Vitamin D—crucial for bone health, immune function, and hormone regulation—is deficient worldwide, affecting around half the population, particularly women. The study aims to determine the prevalence and risk factors of vitamin D deficiency and hypovitaminosis D in non-pregnant women in Lebanon. Methods: A national cross-sectional survey sampled households across Lebanon, covering 2803 non-pregnant women aged 15 to 49. Demographic information and dietary habits were collected, and anthropometric measurements and serum analyses, including 25-hydroxyvitamin D (25(OH)D) concentrations, were conducted. Multivariable Poisson regressions were constructed to calculate the adjusted prevalence ratio (aPR) for vitamin D deficiency and hypovitaminosis D of variables. **Results**: The prevalence of vitamin D deficiency (<30 nmol/L) among non-pregnant women in Lebanon was 37.9%, while 69.2% had hypovitaminosis D (<50 nmol/L). Wearing a veil (hijab) was identified as the most significant risk factor for both vitamin D deficiency (aPR = 3.76) and hypovitaminosis D (aPR = 1.47). Additionally, olive skin and dark skin were both associated with an increased prevalence of vitamin D deficiency (olive skin: aPR = 1.14; dark skin: aPR = 1.28), while only dark skin color was associated with hypovitaminosis D (aPR = 1.10). In contrast, protective factors against vitamin D deficiency and hypovitaminosis D included daily sun exposure exceeding one hour (aPR = 0.83–0.91) and vitamin D supplementation (aPR = 0.30–0.55). Anemia, folate deficiency, and vitamin B12 deficiency were significantly associated with a higher prevalence of vitamin D deficiency, hypovitaminosis D, or both. BMI was not significantly associated with vitamin D deficiency; however, women with underweight (aPR = 1.13) and obesity (aPR = 1.12) exhibited a higher prevalence of hypovitaminosis D. **Conclusions**: Vitamin D deficiency and hypovitaminosis D affect a significant portion of non-pregnant women in Lebanon, with veiling (hijab wearing), limited sun exposure, and lack of supplementation as primary risk factors. Future work should focus on tailoring recommendations for vitamin D supplementation, sun exposure, and food fortification to effectively address the diverse risk factors in the population.

## 1. Introduction

Vitamin D is a vital fat-soluble vitamin and an active prohormone involved in many body metabolisms, such as bone density, immune function, and hormone regulation. Recent studies have revealed new insights into the involvement of vitamin D in obesity, neuronal diseases, cancer, diabetes, cardiovascular diseases, multiple sclerosis, fertility, and many others [1,2,3].

Hypovitaminosis D, also referred to as suboptimal levels of vitamin D, has been reported worldwide and is considered a public health concern. In 2023, a pooled analysis of 7.9 million people aged 1 year or older from 81 countries showed that around 48% had vitamin D insufficiency or deficiency, considering serum 25-hydroxyvitamin D (25(OH)D) concentrations less than 50 nmol/L (≈20 ng/mL), with a higher prevalence among females [4]. Due to food fortification [5,6,7] and the increase in dietary supplement consumption over the years [8,9,10,11], recent data have confirmed improvements in vitamin D status in many countries such as Canada [12], Finland [5], and India [13]. However, significant data gaps continue to be a major obstacle to fully understanding the situation in several low- and middle-income countries [14].

Even though vitamin D is synthesized when the body is exposed to ultraviolet (UV) B radiation, serum 25(OH)D concentrations are reported to be low in countries with abundant sunshine. It has been documented that Middle Eastern countries experience a high prevalence of suboptimal levels of vitamin D [15], with 30% to 90% of the population having concentrations below 50 nmol/L [16].

In Lebanon, data describing the status of vitamin D are limited. Available studies often rely on secondary data from laboratories, or they are based on small sample sizes and restricted to specific geographical areas. Data analysis from medical centers in Lebanon spanning seven years and including more than 150,000 patients revealed a prevalence of 39% of hypovitaminosis D, using the cutoff of 50 nmol/L [17]. However, the primary limitation of this study is that it relies on secondary data analysis from individuals who voluntarily accessed medical centers for blood testing, which introduces potential bias. A more recent community-based study conducted in Beirut on 446 adults reported that 72% of the participants had a suboptimal serum 25(OH)D concentration (<50 nmol/L) [18].

While hypovitaminosis D affects males and females of any age group, women of childbearing age are particularly vulnerable, as suboptimal levels of vitamin D have been associated with adverse maternal and fetal outcomes. During early pregnancy, vitamin D has been shown to regulate the development of the uterus, permitting it to better receive implantation [19]. Later in pregnancy, women with low vitamin D status presented a fourfold increase in the odds of developing severe preeclampsia [20] and a 49% increase in the odds of developing gestational diabetes [21]. A significant relationship was also reported between maternal vitamin D status and fetal health. Mothers in the lowest quartile of 25(OH)D concentrations had two- and 1.5-fold greater risks of having small children for gestational age (SGA) and low birth weight, respectively [22], both indicators of poorer intrauterine fetal growth that increase risks of later-life chronic disease [23].

Various factors have been identified as contributors to total serum vitamin D levels worldwide. Nonetheless, determining country-specific factors remains the most effective approach for understanding a population’s situation and efficaciously addressing its health problems.

The aim of this study was to examine the extent to which established determinants influence vitamin D levels in non-pregnant women 15–49 years of age within the context of Lebanon, as well as to identify additional potential factors. The findings will provide valuable insights for national policymakers regarding effective interventions and enhance the existing body of knowledge on the factors contributing to vitamin D levels.

## 2. Materials and Methods

### 2.1. Survey Design and Participants

Data used in this manuscript stem from the Lebanon Integrated Micronutrient, Anthropometry, and Child Development Survey (LIMA) 2023 [24]. It was designed as a national cross-sectional survey with 10 strata, 8 of which were geographical strata covering the governorates of Lebanon: Akkar, Baalbek-Hermel, Beirut, Bekaa, El Nabatieh, Mount Lebanon, North and South. In addition, one Syrian informal settlement (IS) stratum and one Palestinian camps stratum were created. The sampling universe consisted of all households residing in Lebanon at the time of survey data collection, regardless of nationality and/or ethnicity. Due to the absence of census data, a sampling frame for 8 governorate strata was developed using a hexagon grid, where each hexagon served as an enumeration area (EA), and satellite imagery was used to estimate the population in each EA [24]. For the Palestinian camps stratum, EA lists and maps from the 2017 Population and Housing Census in Palestinian camps and Gatherings in Lebanon (PHCCG-2017) were provided by the United Nations Relief and Works Agency for Palestine Refugees in the Near East (UNRWA) [25]. For the Syrian IS stratum, updated settlement lists for all governorates were provided by the United Nations High Commissioner for Refugees (UNHCR). A two-stage sampling procedure was used to select EAs and, subsequently, households. In the first stage of sampling, EAs were selected with probability proportional to size, and in the second stage, households were selected with equal probability. In each of the selected households, all preschool children 0–59 months, adolescent girls 10–19 years, and pregnant women (of any age) were recruited. Non-pregnant women 20–49 years were included from every second household as a larger number of individuals were expected in this target group.

**Figure 1:**
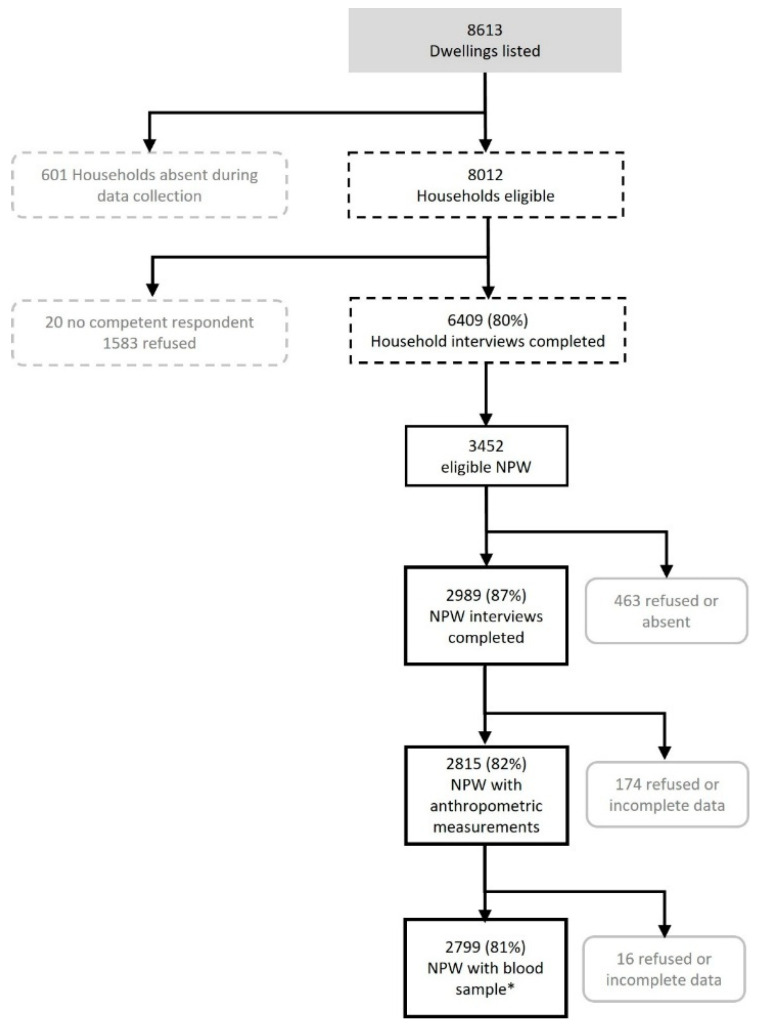
Flow diagram of participants’ response rate. NPW: non-pregnant women of 15 to 49 years old. This chart was adapted from the initial LIMA report [24]. * The sample size varied for each biochemical parameter. The reported sample size corresponds to the total number of samples analyzed for the complete blood count, which was the first test conducted.

Nationally, the LIMA selected 464 EAs, with different numbers in each of the strata, due to expected differences in response rates, household size, and household composition between the strata. A flow chart of data collection and participation rate for non-pregnant women (NPW) is presented in Figure 1.

### 2.2. Data Collection

Listers and data collectors (interviewers, anthropometrists, and phlebotomists) were extensively trained on the survey protocol before the beginning of the fieldwork that took place in July 2023 and ended in November of the same year. The trained personnel were retained on the basis of their performances and post-test grades.

#### 2.2.1. Questionnaires

Two questionnaires were administered by trained interviewers in the selected households: a household questionnaire and an individual questionnaire. The household questionnaire was administered to the head of the household to collect information on household socioeconomic status and classify households into quintiles according to the wealth index of the World Bank [26]. In addition, the Household Food Insecurity Access Scale (HFIAS) was used to assess food security situations and categorize households into four different levels (food secure and mild, moderately, and severely food insecure) [27].

Based on the household roster, eligible non-pregnant women of reproductive age (15–49 years old) were directly recruited, and individual questionnaires specifically developed for this population were administered. These questionnaires were linked to their corresponding household questionnaire and gathered data on education, vitamin and mineral supplement consumption, pregnancy and lactation, sun exposure, and tobacco smoking. The dietary diversity was also evaluated using the Food and Nutrition Technical Assistance (FANTA) methodology [28]. This approach evaluates the consumption of various food groups by women over the preceding day or night. Achieving minimum dietary diversity is defined as the consumption of at least five distinct food groups during this period. Interview data were collected using KoboCollect version v2022.4.4 on tablet computers. Both questionnaires in English and Arabic are available in the appendix section of the LIMA survey report [24].

#### 2.2.2. Anthropometric and Blood Pressure Measurements

Anthropometric measurements were performed on all survey participants. Weight, height, and waist circumference were measured using a medical scale (SECA, Hamburg, Germany), a stadiometer (UNICEF #S0114520, Copenhagen, Denmark), and a measuring tape (SECA 201, SECA, Hamburg, Germany), respectively. Each anthropometric measurement was carried out twice, and the average was recorded. Before blood samples were taken, blood pressure was measured three times within 10 min on the left arm using a semi-automatic blood pressure device (OMRON 300, OMRON Healthcare, Kyoto, Japan), and the average of the three measurements was recorded.

#### 2.2.3. Biological Fluids Collection and Analyses

Blood was collected via venipuncture from the antecubital vein into 5 mL serum-separating tubes (SSTs) and 3 mL plasma ethylenediaminetetraacetic acid (EDTA) tubes (Becton Dickinson, Franklin Lakes, NJ, USA). Immediately after blood collection, tubes were gently inverted and stored in the dark at +4 °C to +8 °C until further processing.

Blood samples were transported on the same day to the American University of Beirut Medical Center (AUBMC) laboratory, which is accredited by the College of American Pathologists, for processing and analysis.

Complete blood count (CBC) was measured in EDTA whole blood samples using a COULTER UNICEL DxH SER (Beckman Coulter, Brea, CA, USA). Glycated hemoglobin (HbA1c) was then measured in the same tube using a CAPILLARYS analyzer (Sebia, Lisses, France). Moreover, plasma was analyzed for retinol-binding protein (RBP), serum ferritin (SF), soluble transferrin receptor (sTfR), C-reactive protein (CRP), and α1-acid glycoprotein (AGP) at the VitMin Lab (Willstätt, Germany) using a sandwich enzyme-linked immunosorbent assay (ELISA) method [29].

The SSTs were centrifuged upon receipt, and serum folate, vitamin D (25(OH)D), and vitamin B12 concentrations were analyzed on a Cobas 8000 (e801) (Roche Diagnostics GmbH, Mannheim, Germany). Serum triglyceride, total cholesterol, and high-density lipoprotein (HDL)-cholesterol were measured using Cobas 8000 (C702).

### 2.3. Indicators and Thresholds

Table 1 below presents the cutoffs for biomarkers discussed in this paper for non-pregnant women, along with their corresponding classifications. Various cutoffs are used to define vitamin D status. In this study, the reference values established by the Institute of Medicine (IOM) were adopted, classifying vitamin D deficiency as a serum concentration of less than 30 nmol/L (12 ng/mL) and insufficiency as levels ranging between 30 and 50 nmol/L (12 and 20 ng/mL) [30]. To improve clarity and facilitate the readability of the manuscript, the term “deficiency” or “deficient” will be used to refer to women with serum 25(OH)D levels below 30 nmol/L (12 ng/mL), and “hypovitaminosis D” will refer to women with serum 25(OH)D levels below 50 nmol/L (20 ng/mL).

### 2.4. Data Management and Statistical Analyses

Data analysis was performed using Stata version 18. Statistical weights were applied to all data to account for the unequal probability of selection in the 10 strata. The distribution of serum 25(OH)D values was checked using the Shapiro–Wilk W test, and data are presented as weighted medians with interquartile ranges for skewed data. All other continuous variables (risk factors) were categorized into subgroups (e.g., quantiles or other standard categorizations). For all categorical variables, weighted proportions were calculated to determine the prevalence of various outcomes. Missing cases were handled by listwise deletion. The statistical precisions of all prevalence estimates were assessed using 95% confidence intervals, which were calculated to account for the complex sampling used in this survey, including the cluster and stratified sampling. The chi-square test of independence was used to examine the statistical differences in vitamin D deficiency or hypovitaminosis D prevalence between subgroups for each risk factor.

Risk factors identified in the bivariate chi-square analysis as being associated with vitamin D deficiency or hypovitaminosis D (*p*-value < 0.200) were included in the respective multivariate Poisson regression. When variables were found to be collinear (variance inflation factor > 2), only one of the collinear variables was typically included in the model to avoid redundancy and multicollinearity issues. To investigate the effect of sun exposure and vitamin D supplementation on vitamin D concentration, a linear regression analysis was run using the natural logarithm of vitamin D concentration, the Box–Cox transformation of the daily sun exposure time, to achieve normally distributed data (Shapiro–Wilk W > 0.97), with supplementation as a factor variable. Statistical significance was set at a *p*-value of 0.05.

### 2.5. Ethical Considerations

Ethical approval for the study was obtained from the American University of Beirut Institutional Review Board (BIO-2023-0106, 7 July 2023). Data collection was conducted according to the approved protocol. Written consent was obtained from all the adult participants. Caregivers provided written informed consent for their participating adolescent girls who were also asked to provide written assent. Participants who were diagnosed with malnutrition, hypertension, severe anemia, or diabetes were referred to a health facility.

## 3. Results

A total of 2803 non-pregnant women had their serum analyzed for vitamin D, 71.6% of whom were Lebanese, 25% were Syrian, 2% were Palestinian, and approximately 1.2% were of other nationalities. The median (IQR) age of the study participants was 33.5 (23.3, 40.7) years. More than half the sample population (54.2%) was described as having an olive skin color, and around 58% of women wore the hijab. By hijab wearing or veiling, we refer to women who wear modest clothing along with a head covering, leaving only the face, hands, and sometimes feet exposed to sunlight. More baseline demographic characteristics are detailed in Table 2.

**Figure 2:**
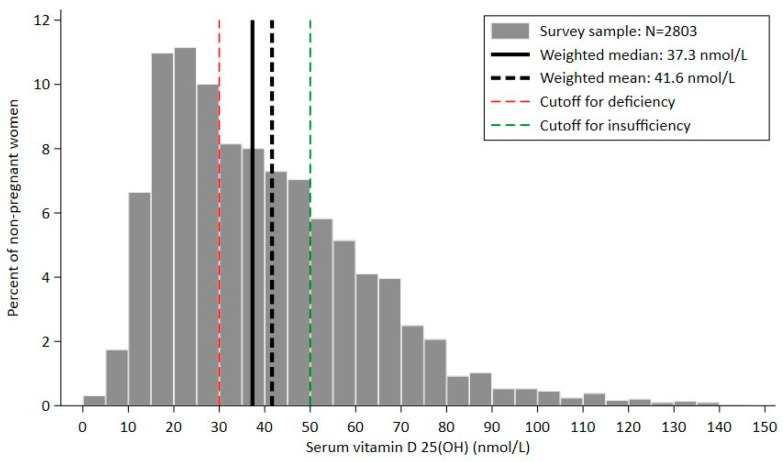
Distribution of serum 25-hydroxyvitamin D (25(OH)D) levels in non-pregnant women living in Lebanon.

The population distribution was skewed to the right, indicating a high prevalence of low serum 25(OH)D (Figure 2). Values ranged between 7.5 nmol/L and 227.5 nmol/L, with a weighted median of 37.3 nmol/L. Adequate levels of vitamin D (>50 nmol/L) were found among 30.8% of women, leaving 69.2% with suboptimal levels, 37.9% of whom had serum concentrations below 30 nmol/L (vitamin D deficient).

Histogram showing the distribution of serum 25(OH)D levels (nmol/L) among 2803 non-pregnant women aged 15 to 49 years old in Lebanon. The green dashed line is the cutoff for vitamin D insufficiency (50 nmol/L), and the red dashed line is the cutoff for deficiency (30 nmol/L). The thick black solid line represents the weighted median value of the population (37.3 nmol/L), and the thick black dashed line represents the weighted mean value of the population (41.6 nmol/L).

### 3.1. Vitamin D Deficiency and Hypovitaminosis D Risk Factors

Supplementary Tables S1–S3 present bivariate analyses of the various indicators associated with vitamin D deficiency and hypovitaminosis D examined in this study. Vitamin D deficiency or hypovitaminosis D was seen in greater proportions among women who had darker skin colors, the least sun exposure time (less than 1 h), and who wore hijab but no sunscreen and took no supplements. In addition, vitamin D deficiency and hypovitaminosis D were lower among Lebanese compared to non-Lebanese women. An inverse relationship was shown between the prevalence of vitamin D deficiency and age, wealth quintile, educational level, and food security access. More details of the results of the bivariate analysis are presented in the Supplementary Materials.

All variables with a *p*-value < 0.2 in the bivariate analyses were included in the multivariate analysis to ensure inclusivity and capture potential risk factors.

A multivariate logistic regression model was used to study factors that significantly affect the prevalence of vitamin D deficiency and hypovitaminosis D among non-pregnant women in Lebanon. All factors with a *p*-value of <0.2 in the bivariate analyses were included in the multivariate analysis. Central obesity and iron deficiency anemia were dropped from the multivariate model because they were collinear with BMI and anemia, respectively.

The model confirmed the significance of several factors known to influence the prevalence of vitamin D deficiency and hypovitaminosis D to different extents (Table 3).

These components include skin color, extensively and constantly covering body parts (hijab wearing), daily sun exposure, use of sunscreen, and vitamin D supplementation. The skin color gradient showed a clear effect on vitamin D status. Compared with lighter skin tones (very white/white), darker skin tones (olive and dark/very dark) were linked to a greater risk of both deficiency (adjusted prevalence ratio (aPR) = 1.14 for olive and aPR = 1.28 for dark/very dark) and hypovitaminosis (no effect for olive tones and aPR = 1.10 for dark/very dark). Compared with other women, women who wore the hijab had a 3.8-fold greater risk of vitamin D deficiency and a 1.47-fold greater risk of hypovitaminosis D compared to others. Also, a sun exposure time of 1 to 2 h correlated with increased vitamin D levels and a 10 to 16% reduction in hypovitaminosis D and vitamin D deficiency, respectively. However, extended sun exposure did not provide significant additional benefits. In parallel, while vitamin D supplementation had the most important preventative effect on both deficiency (70% lower risk) and hypovitaminosis (45% lower risk), this model did not demonstrate a significant effect of multivitamin supplementation on either outcome. The use of sunscreen was associated with a decrease in vitamin D deficiency and hypovitaminosis by 11 to 18%. This effect might not be due to sunscreen itself but rather to the characteristics and behavior of sunscreen users versus compared to non-sunscreen users. A weighted post hoc analysis revealed that hijab wearers were more represented among non-sunscreen users (63%) than among sunscreen users (51%) (Chi-sq = 35.6; F (1,418) = 11.1; *p*-value < 0.001). Additionally, vitamin D supplementation was more common among sunscreen users (23.3%) compared to non-users (17.2%) (*p*-value = 0.037). Furthermore, sunscreen use showed a strong positive correlation with household wealth quintile and food security levels (*p* < 0.001), suggesting that socioeconomic factors may also play a role in the observed differences.

The model also reveals that, compared with the older age group (40–49 years), the youngest group (15–19 years) presented the highest risk of vitamin D deficiency. As age increased, the risk of both deficiency and hypovitaminosis gradually decreased. Additionally, individuals in the lowest wealth quintiles tended to experience heightened risks of hypovitaminosis (13% to 17%), whereas the impact of wealth on deficiency was more pronounced for the second and middle quintiles (21% to 28%). Moreover, a higher educational level provided some protective effects against vitamin D deficiency but not hypovitaminosis. Interestingly, this model did not demonstrate any significant effect of household food security access, minimum dietary diversity, tobacco smoking, or breastfeeding on vitamin D status.

With respect to the metabolic and nutritional, several indicators showed association with vitamin D status. Anemia increased the risk of vitamin D deficiency by 14%. Furthermore, the risk of hypovitaminosis D was augmented by 13 and 12% in underweight and obese individuals, respectively (compared with healthy and overweight individuals) and by 10% in women with folate deficiency. Low HDL cholesterol and vitamin B12 deficiency were also risk factors for both vitamin D deficiency and hypovitaminosis. The only metabolic disturbance that had a protective effect against vitamin D deficiency was elevated serum triglyceride, offering a 13% decrease in risk.

### 3.2. Hijab Wearing and Vitamin D Supplementation: Key Predictors of Vitamin D Status

**Figure 3:**
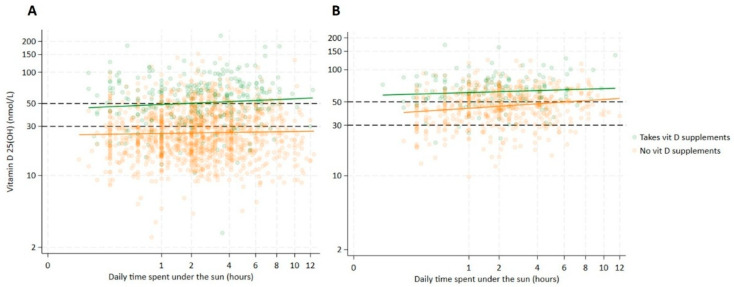
Scatter plot of serum 25-hydroxyvitamin D (25(OH)D) and daily sun exposure time by patterns of vitamin D supplement use among non-pregnant women.

Figure 3 presents scatter plots illustrating the most significant predictors of serum 25(OH)D concentrations: hijab use and vitamin D supplementation. The plots show the relationship between daily sun exposure duration and serum 25(OH)D levels across two groups of non-pregnant women: those wearing hijab (A) and those not wearing hijab (B), stratified by their vitamin D supplementation status. Among women wearing hijab, daily sun exposure time was not a predictor of vitamin D concentration (*p*-value = 0.068). When women did not take vitamin D supplementation, despite prolonged sun exposure, vitamin D concentrations remained below the deficiency threshold (<30 nmol/L). However, women wearing hijab and taking vitamin D supplementation had higher vitamin D concentrations than those who did not (*p*-value < 0.001), and prolonged sun exposure (>2 h daily) resulted in vitamin D concentrations reaching sufficiency levels (>50 nmol/L). On the other hand, among women not wearing hijab, sun exposure was a significant predictor (*p*-value = 0.001) of serum 25(OH)D concentrations, and both in the presence or absence of vitamin D supplementation, vitamin D concentrations were consistently higher than those observed in the corresponding groups wearing a hijab. Notably, vitamin D levels were particularly elevated among non-veiled women taking supplementation but reached sufficiency after prolonged sun exposure (>6 h) also in women not taking supplements.

Figure 3 presents scatter plots showing the distribution of serum 25(OH)D levels (nmol/L) and daily sun exposure time in hours among non-pregnant women aged 15 to 49 years old, (A) wearing a hijab (*N* = 1605) and (B) not wearing a hijab (*N* = 706), respectively, in Lebanon. The dashed lines are the cutoff for vitamin D insufficiency (50 nmol/L) and vitamin D deficiency (30 nmol/L). Green dots and the corresponding best-fit line represent women taking vitamin D supplements, and orange dots and the corresponding best-fit line represent women not taking supplements.

## 4. Discussion

This study presents the first national estimates of vitamin D status and its determinants among reproductive-age women living in Lebanon. Its comprehensiveness with respect to the interaction of vitamin D with micronutrients and nutritional biomarkers constitutes an important contribution to knowledge.

The prevalence of vitamin D deficiency in the country is 37.9%, using a reference value of 30 nmol/L. When a cutoff of 50 nmol/L was applied, the prevalence rises to 69.2%. Consequently, only 30.8% of women are found vitamin D sufficient. Our findings are comparable to those of a small study in women living in Beirut [18]. This population proportion is higher than the global numbers, which is estimated to be 53.3% among females, with 50 nmol/L used as a reference [4]. A lower prevalence of hypovitaminosis D was reported among females in Oman (57.7%) [43] and most countries in Europe [44], while in Basrah-Iraq (66.5%) [45], Turkey (64.7%) [46], and Egypt (70.9%) [47], the prevalence was similar. Only in Jordan were the proportions of women with suboptimal levels of vitamin D higher (81.3%) [48]. It is worth noting that these studies do not necessarily share the exact age range, vitamin D cutoffs, or analytical methods, which makes comparisons difficult [49].

### 4.1. Synthesis-Related Risk Factors

Circulating vitamin D levels result from a combination of dietary intake (contained natively in foods or added through food fortification), supplementation, and sun exposure. Vitamin D is synthesized in the skin from 7-dehydrocholesterol in a two-stage process. UVB radiation from sunlight initiates the conversion of 7-dehydrocholesterol into previtamin D_3_, which undergoes thermal isomerization to form vitamin D_3_. Transported by the vitamin D-binding protein (gene DBP), vitamin D is then hydroxylated by the 25-hydroxylase (gene CYP2R1) in the liver and to the kidney (and other tissues) to be metabolized by the 25-OH-d-1α-hydroxylase (gene CYP27B1) to its active form 1,25-dihydroxycholecalciferol [50].

Data collection for this study was conducted over 4 months, spanning the summer and fall. The analysis revealed a statistically significant positive relationship, with vitamin D levels increasing by 1.25 nmol/L per month from July to mid-November. Despite statistical significance, the observed increase was considered to have minimal clinical relevance. Consequently, seasonal variability was not incorporated into the analysis in this paper.

Lebanon is a Middle Eastern country with about 300 days of sunshine per year. It records an estimated UV Index ranging from 11 in July to 5 in November in Beirut. Lebanon is a small country with relatively uniform climatic conditions, and the UV Index does not vary significantly across different regions. Even in December, when the UV Index is at its lowest, it remains in the moderate range, meaning that vitamin D synthesis is still possible with extended sun exposure. Despite this, more than two-thirds of the women in this country show a suboptimal vitamin D status. This paradoxical situation necessitated further analyses to uncover the underlying factors.

The association between skin color and vitamin D status is well-documented [51,52]. In regions with abundant sunlight, populations often have darker skin tones, which affects the skin’s ability to photochemically convert cholesterol precursors into vitamin D. This association is mediated by melanin pigments. Melanin absorbs UV radiation and diminishes its effectiveness in stimulating vitamin D synthesis. While melanin acts as a protective mechanism against UV-induced damage, it inadvertently contributes to blunting vitamin D synthesis and consequently increasing the risk for vitamin D deficiency [53]. Our results show a clear inverse association between skin pigmentation and serum 25(OH)D levels: women with darker skin exhibited lower serum concentrations of vitamin D and a greater prevalence of hypovitaminosis D. In support of these findings, a study comparing the effect of sun exposure in White and South Asian participants on the serum levels of 25(OH)D reported higher levels in White compared to South Asian participants after sun exposure (10.5 ng/mL increment versus 4.3 ng/mL) [54]. Counterintuitively, our study found an aPR of 0.82 and 0.89 for sunscreen use. In fact, the negative effect of sunscreen on vitamin D status could have been overruled by other factors such as veiling and skin color. Our data show a clear trend between the application of sunscreen and skin color. A greater proportion of sunscreen users had lighter skin tones (37.5%), followed by olive color (33.7%) and darker skin tones (26.8%). Nevertheless, this difference did not reach statistical significance.

Within the same context of sun exposure, our survey reveals a significant association between wearing a hijab (coverage of the whole body excluding the face and hands) and vitamin D deficiency. Compared with those who did not wear a hijab, women who wore a hijab had a 3.8-fold greater prevalence of vitamin D deficiency and a 1.5-fold great prevalence of hypovitaminosis D. Body coverage constitutes a barrier to effective cutaneous vitamin D synthesis, and hijab-wearing consistently keeps levels of vitamin D below the cutoffs when no supplementation is taken, as shown in the scatter plot. Since sun exposure has no impact on veiled women, the small protective effect of sun exposure observed in this study (PR = 0.84 for deficiency and PR = 0.9 for hypovitaminosis) would likely have been significantly larger if the study had been conducted exclusively among non-veiled women. In the summer season, Mishal reported a similar risk of a 1.8-fold increase in the prevalence of hypovitaminosis D in a small sample of Jordanian women with hijab. This same paper highlighted a 2.7-time risk increment among women wearing the niqab (total coverage of the whole body, including hands and face) [55]. Another study also indicated an adjusted odds ratio of 1.5 to 1.7 for vitamin D deficiency among women wearing a type of coverage, considering a threshold of 75 nmol/L [56]. Intriguingly, a paper by Gannage-Yared et al. investigating risk factors for vitamin D inadequacy among Lebanese postmenopausal women reported different risk factors between women belonging to a Muslim versus Christian community. In multivariate models and aside from supplementation, dress code imposing arms coverage was a predictor of vitamin D inadequacy among Muslims, while high BMI and low educational level were predictors among Christians [57].

### 4.2. Intake-Related Risk Factors

In the absence of a UV stimulus, vitamin D must be obtained from external sources (diet or supplements). Vitamin D is a fat-soluble vitamin; while it is well absorbed from food and supplements, vitamin D absorption can be influenced primarily by factors affecting fat absorption [58]. Interestingly, in this study, food-related indicators such as food security level and minimum dietary diversity show no effect on vitamin D status according to the multivariate models, while vitamin D supplementation was found to reduce the prevalence of deficiency by 70%. However, the efficiency of vitamin D supplementation in reducing the prevalence of hypovitaminosis is approximately 36% lower, hinting at the presence of some heterogeneity in the population in response to supplementation, which has been further confirmed in Figure 3. Indeed, in their randomized controlled trial, Barry et al. revealed that after 1 year of vitamin D supplementation, serum vitamin D levels fluctuated differently with different genetic variants in the vitamin D pathway genes [59]. Another plausible explanation for the different effects of supplementation on the severity of hypovitaminosis could be related to the dosage required to treat the condition. Vitamin D amounts needed to correct deficiency exceed the ones needed for hypovitaminosis. Since vitamin D is absorbed by simple passive diffusion, which highly depends on the concentration gradient, a higher dosage or a lower status results in greater absorption. Indeed, the slope representing vitamin D supplementation among veiled women in Figure 3 is greater than that among non-veiled women.

### 4.3. Weight-Related Risk Factors

The interplay between vitamin D metabolism and obesity has been a recent topic of interest. Our data show lower serum 25(OH)D levels in women with a BMI < 18.5 and ≥30 kg/m^2^, compared to levels in normal and overweight women. Numerous studies have confirmed an association between increased adiposity and vitamin D deficiency. An analysis of 16,375 participants showed that baseline serum concentrations of vitamin D were incrementally lower at higher BMIs (32.4 ng/mL for a healthy BMI, 29 ng/mL for 30.0 ≤ BMI ≤ 34.9 kg/m^2^, and 28 ng/mL for BMI ≥ 35.0 kg/m^2^) [60]. No difference from healthy BMI was reported for underweight individuals. Interestingly, the same paper showed that 2 years of vitamin D supplementation resulted in a significantly lower increase in serum vitamin D in participants with higher BMI [60]. These results have been extensively discussed in the literature [61,62,63]. Walsh et al. proposed the concept of vitamin D dilution, which explains the lower status in people with obesity [64]. While the amount of vitamin D is the same, the larger body/tissue size and blood volume may result in a lower concentration. Inline, Wortsman et al. hypothesized that vitamin D can be sequestered in the adipose tissue, which decreases its bioavailability [65]. The authors demonstrated that despite the increase in the surface area of the skin, hence UV exposure, the increment of vitamin D serum levels was 57% lower in obese than non-obese individuals. They attributed these findings to lower levels of circulating vitamin D, since the percentage of 7-dehydrocholesterol in the skin that was converted to vitamin D was not significantly different between the two groups. In this case, adipose tissue serves as a reservoir of vitamin D in people with obesity, making it less readily available in the bloodstream. A systematic review and meta-regression analysis reported an increase in 25(OH)D levels accompanying weight loss [66]. Interestingly, the upsurge in the serum vitamin D concentration upon weight loss is proportional to weight loss itself, reflecting vitamin D mobilization from tissues. A 3.7-fold greater increase was observed in women who lost 15% or more of weight than in those whose weight loss was less than 5% [67].

Adipose tissue is not only a storage place but also an endocrine system whose secretions affect vitamin D metabolism. In vivo studies revealed the involvement of leptin, an adipocyte-made hormone, in changing renal 25-OH-d-1α-hydroxylase (CYP27B1) mRNA levels, decreasing the concentration of the vitamin D active form [68]. Our results align with this hypothesis since leptin secretion is proportionally related to the number of adipocytes, which in turn increases with BMI. Our data indicate that while being overweight did not significantly elevate the prevalence of hypovitaminosis, being obese, which is accompanied by higher leptin levels, was associated with a 12% increase. All the abovementioned theories were proven right with studies showing improved vitamin D status with weight loss [67].

Nevertheless, the causality of the problem remains inconclusive. A systematic review concluded a potential contribution of vitamin D deficiency to the occurrence of obesity [69]. Plausible mechanisms through which vitamin D might contribute to obesity include parathyroid hormone (PTH) [70] and vitamin D receptor (VDR) [71]. Through different molecular pathways, the drop in vitamin D levels might promote fat accumulation. In support, a double-blind clinical trial on 20- to 40-year-old overweight and obese women noted a decrease in weight, waist circumference, and hip circumference 6 weeks after starting vitamin D supplementation of 50,000 IU/week [72].

Both low and high body weights can negatively affect vitamin D status through different mechanisms. Underweight, in this study, had a prevalence ratio close to the one of obesity, with an increase in the prevalence of hypovitaminosis D by 13%. It is safe to postulate that this association is attributable to low intake and absorption of vitamin D. In a systematic review and meta-analysis on children, serum vitamin D was inversely related to undernutrition, where the risk estimate of wasting was reported to be 1.3 (1.04, 1.63) [73]. Nevertheless, some studies reported no differences in serum vitamin D levels or the prevalence of vitamin D inadequacy between individuals with a healthy BMI and underweight individuals [60], while others merged people with a normal BMI with those who were underweight due to their small sample size [74].

### 4.4. Biochemical Risk Factors

A small yet significant association was found between HDL cholesterol and vitamin D, whereby low serum HDL increases the prevalence of hypovitaminosis D by 6% and deficiency by 14%. However, the causal relationship of this association remains unclear. In agreement, a recent meta-analysis reported a pooled effect size of 0.08 (95% CI: 0.01, 0.15) of vitamin D supplementation on serum HDL [75]. In addition, laboratory databases from a hospital in North Lebanon, presenting results of 8658 subjects, affirmed a positive association between serum levels of 25(OH)D and HDL cholesterol [76]. A tight relationship has been described between endothelial function and inflammation on the one hand and HDL cholesterol on the other hand, explaining potential working areas of vitamin D [77]. Another postulated mechanism involves lipoprotein lipase (LPL). Incubating murine preadipocytes with the active form of vitamin D increases mRNA expression and activity of LPL [78], which mediates triglyceride hydrolysis and clearance and increases HDL cholesterol [77]. While the latter mechanisms explain our HDL results, they do not account for why elevated serum triglycerides are protective against vitamin D deficiency, a finding that contradicts existing literature reporting no [79] or positive associations between serum vitamin D deficiency and triglyceride concentrations [80,81].

Our data also show associations between vitamin D status and anemia, folate deficiency, and vitamin B12 deficiency. Different micronutrient deficiencies often coexist due to poor intake, complicating the interpretation of their associations. While this has long been believed to be the case for the relationship between hypovitaminosis D and anemia, some insights have linked this relationship to inflammation. With its anti-inflammatory properties, vitamin D has been shown to decrease monocytic secretion of interleukin-1β (IL-1 β) and IL-6, which can stimulate hepcidin mRNA expression [82]. This explanation suggests that existing anemia might be metabolically linked to underlying inflammation. In addition, the active form of vitamin D was shown to increase the mRNA expression of erythropoietin receptor, the mediator of red blood cell production [83]. The link between vitamin D and anemia might also be mediated by folate or vitamin B12, key factors in nutritional anemia. Correlations between vitamin D, folate, and B12 deficiencies have been observed [84,85] and investigated. While it is fair to assume that vitamins D and B12 deficiencies just coincide since both have similar food sources such as eggs, fish, and organ meats and have been associated with household wealth, scientific reports have highlighted phenomena through which vitamin D deficiency negatively impacts B12 status. Vitamin D deficiency can diminish vitamin B12 status by affecting epithelial integrity [86], and through decreasing calcium levels, which might compromise B12 absorption, a calcium-dependent process at the terminal ileum level [87]. On the other hand, no direct association has been described between vitamin D and folate. However, an indirect association is plausible given the involvement of both vitamins in B12 metabolism. Due to the close relationship between vitamin B12 and folate in the methionine synthase reaction, functional folate deficiency can occur under conditions of vitamin B12 deficiency. This phenomenon, known as methyl trapping, leads to a decrease in tissue levels of some folate-dependent coenzymes [88]. While this pathway affects folate activity, no record has shown an effect on serum levels.

### 4.5. Demographic Risk Factors

Demographic factors are not direct causes of hypovitaminosis D but are key in determining its risk. They serve as underlying contributors that influence a population’s vulnerability to low vitamin D levels. In this study, younger women presented with lower serum levels and a greater prevalence of hypovitaminosis than older women did. In a cross-sectional analysis based on NHANES data, including 7855 females aged 20 years or older, vitamin D concentrations and age were positively correlated. Even after adjusting for confounders, older age was still seen as a protective factor against deficiency [89]. This has been attributed to faster metabolism and the role of vitamin D in decelerating the aging process, a process that is diminished with age [89]. Our study further affirmed that women from the lower wealth quintiles (lowest until middle) are at greater risk of vitamin D deficiency and/or hypovitaminosis. This finding aligns with previous research indicating that inadequate household income, low socioeconomic status, or poverty—different terms for the same underlying risk factor—are associated with this increased risk. Nationality was not included in the multivariate model, since it is likely to be an indirect indicator of a lower wealth index and body coverage practices. The data suggest that women of Syrian nationality are subjected to an increased risk of vitamin D deficiency and hypovitaminosis D. In fact, around 80% of Syrian women belong to households of the lowest two wealth quintiles, and more than 88% of them are veiled. A lower educational level is associated with an 18% increase in the risk of deficiency, a finding that aligns with existing literature [90,91].

This study provides valuable insights into the vitamin D status of women of reproductive age living in Lebanon. The large sample size, high response rate and representativeness of the sample ensure the generalizability of its findings. However, the main limitation of this study lies in its design as a cross-sectional study, which prevents causal interpretation between variables. Additionally, data collection took place mostly during the summertime when sun exposure among unveiled women was significantly greater than that at other times of the year.

## 5. Conclusions

A significant proportion of non-pregnant women in Lebanon are affected by hypovitaminosis D, a condition that may have far-reaching consequences for their health. The primary risk factor among this population is wearing a veil (hijab), and the factors offering the greatest protection are sun exposure and vitamin D supplementation. In a context similar to Lebanon, where the population’s risk factors are heterogeneous, addressing vitamin D deficiency and hypovitaminosis D requires carefully tailored strategies. For non-veiled women, increasing sun exposure may be an effective recommendation. However, for veiled women, where sun exposure has no impact on serum 25(OH)D levels, supplementation is essential. Additionally, implementing food fortification programs can provide broad, population-wide benefits, ensuring that everyone, regardless of lifestyle or risk factors, has access to sufficient vitamin D. National strategies should also support awareness campaigns to improve public understanding of vitamin D and its critical role in maintaining good health. Future work should focus on tailoring recommendations for vitamin D supplementation, sun exposure, and food fortification to effectively address the diverse risk factors in the population. Further studies are needed to evaluate the feasibility and effectiveness of these interventions to ensure sustainable strategies for preventing vitamin D deficiency.

## Figures and Tables

**Table 1:** Cutoff points and classifications for biomarkers in non-pregnant women.

Indicator	Condition	Cutoffs
Blood biomarkers		
25(OH)D [30]	Hypovitaminosis D Vitamin D insufficiency Vitamin D deficiency	<50 nmol/L (20 ng/mL) 30 nmol/L (12 ng/mL)–50 nmol/L (20 ng/mL) <30 nmol/L (12 ng/mL)
Hemoglobin ^1^ [31]	Anemia	<120 g/L
Retinol-binding protein ^2, 3^	Vitamin A deficiency	<0.62 µmol/L
Plasma ferritin ^3^ [32]	Iron deficiency	<15 µg/L
α1-acid-glycoprotein [33]	Convalescent inflammation	>1 g/L
C-reactive protein [33]	Early or acute inflammation	>5 mg/L
Folate [34]	Folate deficiency	<4.4 ng/mL (<10 nmol/L)
Vitamin B12 [35]	Vitamin B12 deficiency	<203 mg/L (150 pmol/L)
Zinc [36]	Zinc deficiency	Morning sample in a fasting state: <70 μg/dL Morning sample in a non-fasting state: <66 μg/dL Afternoon sample in a non-fasting state: <59 μg/dL
HbA1c [37]	Elevated HbA1c Pre-diabetes Diabetes	≥5.7% (39 mmol/mol) 5.7–6.4% (39–47 mmol/mol) ≥6.5% (48 mmol/mol)
Triglycerides [38,39]	Hypertriglyceridemia	≥18 years: Fasting: ≥150 mg/dL (1.69 mmol/L) Non-fasting: ≥200 mg/dL (2.26 mmol/L) <18 years: Fasting: ≥90 mg/dL (1.02 mmol/L) Non-fasting: ≥130 mg/dL (1.47 mmol/L)
Total cholesterol [38,39]	Hypercholesterolemia	≥18 years: ≥240 mg/dL (6.2 mmol/L) <18 years: ≥200 mg/dL (5.17 mmol/L)
HDL-cholesterol [38,39]	Low HDL-cholesterol	≥18 years: <50 mg/dL (1.3 mmol/L) <18 years: <40 mg/dL (1.03 mmol/L)
Anthropometry and blood pressure		
Body Mass Index (BMI) ^4^ [40]	Underweight Normal weight Overweight Obesity	<18.5 kg/m^2^ 18.5 to 24.9 kg/m^2^ 25 to 29.9 kg/m^2^ ≥30 kg/m^2^
Blood pressure [41]	Hypertension	Systolic blood pressure ≥ 140 mmHg Diastolic blood pressure ≥ 90 mmHg

^1^ Hemoglobin concentrations were adjusted for elevation and smoking. ^2^ No established deficiency cutoffs have been developed for RBP. Plasma retinol concentration was measured, the threshold for vitamin A deficiency of <0.7 µmol/L [42] was used, and a linear correlation between RBP and plasma retinol was used to estimate a vitamin A deficiency cutoff of 0.62 µmol/L. ^3^ Indicators were adjusted for sub-clinical inflammation using appropriate algorithms. ^4^ Body mass index is calculated: weight (kg)/height (m^2^).

**Table 2:** Basic demographic profile of the sample population.

Characteristics	N ^a^	% ^b^	95% CI ^c^
Total	2803	100.0	
Age group (in years)			
15–19	456	16.4	[14.2, 18.9]
20–29	751	25.3	[22.5, 28.3]
30–39	753	26.6	[23.2, 30.3]
40–49	843	31.8	[28.7, 35.0]
Skin color			
Very white/white	1051	37.1	[34.0, 40.2]
Olive	1529	54.2	[51.7, 56.7]
Dark/very dark	213	8.5	[6.7, 10.6]
Unable to observe	10	0.3	[0.1, 0.6]
Nationality			
Lebanese	2002	71.6	[66.8, 76.0]
Syrian	666	25.0	[20.8, 29.8]
Palestinian	97	2.0	[1.2, 3.3]
Other	29	1.1	[0.6,1.9]
Not known	9	0.2	[0.1, 0.6]
Wealth quintile			
Lowest	677	23.2	[19.6, 27.2]
Second	525	20.3	[16.8, 24.3]
Middle	560	20.0	[17.2, 23.2]
Fourth	526	17.9	[15.1, 21.1]
Highest	515	18.6	[15.0, 22.7]
Educational level			
Basic secondary or less	1488	55.4	[51.6, 59.1]
Complete secondary or more	1306	44.3	[40.6, 48.1]
Not known	9	0.2	[0.1, 0.6]
Household food insecure access			
Secure	897	34.4	[29.8, 39.3]
Mild	272	9.0	[7.0, 11.4]
Moderate	859	28.6	[25.3, 32.2]
Severe	775	28.0	[24.0, 32.4]
Household sanitation			
Inadequate	113	4.1	[2.8, 6.0]
Adequate	2636	93.4	[91.2, 95.1]
Not known	54	2.5	[1.5,4.1]
Wearing hijab			
Yes	1920	58.3	[51.9, 64.4]
No	874	41.4	[35.3, 47.8]
Not known	9	0.2	[0.1, 0.6]

^a^ Ns are the numerators for a specific subgroup. ^b^ Percentages weighted for unequal probability of selection. ^c^ CI = confidence interval, calculated taking into account the complex sampling design.

**Table 3:** Multivariate analysis of known risk factors, demographic, behavioral, metabolic, and nutritional risk factors of vitamin D deficiency and hypovitaminosis D.

Variables	Vitamin D Deficiency		Hypovitaminosis D	
Known Risk Factors	aPR ^a^ [95% CI]	*p*-Value ^b^	aPR ^a^ [95% CI]	*p*-Value ^b^
Skin color				
Very white/white	reference		reference	
Olive	1.14 [1.03, 1.26]	**0.010**	1.05 [1.00, 1.11]	0.062
Dark/very dark	1.28 [1.09, 1.51]	**0.003**	1.10 [1.00, 1.21]	**0.040**
Wearing hijab				
No	reference		reference	
Yes	3.76 [3.05, 4.63]	**<0.001**	1.47 [1.36, 1.58]	**<0.001**
Daily sun exposure				
<1 h	reference		reference	
1–2 h	0.84 [0.74, 0.96]	**0.012**	0.90 [0.84, 0.96]	**0.002**
2–3 h	0.84 [0.74, 0.95]	**0.006**	0.91 [0.85, 0.98]	**0.008**
>3 h	0.83 [0.74, 0.93]	**0.001**	0.90 [0.85, 0.96]	**0.001**
Use of sunscreen				
No	reference		reference	
Yes	0.82 [0.74, 0.91]	**<0.001**	0.89 [0.84, 0.95]	**<0.001**
Vitamin D supplementation				
No	reference		reference	
Yes	0.30 [0.24, 0.39]	**<0.001**	0.55 [0.49, 0.62]	**<0.001**
Multivitamin supplementation				
No	reference		reference	
Yes	0.99 [0.81, 1.22]	0.950	0.93 [0.81, 1.05]	0.231
Demographic characteristics				
Age group in years				
15–19	1.57 [1.37, 1.79]	**<0.001**	1.38 [1.27, 1.49]	**<0.001**
20–29	1.33 [1.17,1.52]	**<0.001**	1.28 [1.19, 1.37]	**<0.001**
30–39	1.17 [1.03, 1.33]	**0.019**	1.12 [1.04, 1.20]	**0.003**
40–49	reference		reference	
Wealth quintile				
Lowest	1.21 [0.99, 1.47]	0.068	1.13 [1.02, 1.25]	**0.016**
Second	1.28 [1.05, 1.56]	**0.015**	1.17 [1.06, 1.28]	**0.002**
Middle	1.25 [1.03, 1.53]	**0.026**	1.04 [0.94, 1.15]	0.484
Fourth	1.15 [0.93, 1.42]	0.190	1.10 [1.00, 1.22]	0.062
Highest	reference		reference	
Educational level				
Basic secondary or less	reference		reference	
Complete secondary or more	0.82 [0.74, 0.92]	**<0.001**	0.96 [0.90, 1.01]	0.127
Household food security access				
Secure	reference		reference	
Mild	1.14 [0.94, 1.38]	0.185	1.02 [0.92, 1.13]	0.751
Moderate	1.00 [0.88, 1.15]	0.974	1.02 [0.95, 1.09]	0.625
Severe	1.10 [0.96, 1.26]	0.175	0.98 [0.91, 1.05]	0.571
Other risk factors				
Minimum dietary diversity				
No	reference		-	-
Yes	0.98 [0.90, 1.07]	0.614	-	-
Tobacco smoking				
No	reference		reference	
Yes	0.95 [0.86, 1.05]	0.282	1.00 [0.95, 1.05]	0.955
Currently breastfeeding				
No	reference		reference	
Yes	1.00 [0.80, 1.25]	0.977	0.95 [0.84, 1.08]	0.431
BMI classes				
Underweight	-	-	1.13 [1.02, 1.25]	**0.016**
Healthy weight	-	-	reference	
Overweight	-	-	1.06 [1.00, 1.13]	0.068
Obese	-	-	1.12 [1.04, 1.20]	**0.001**
Elevated serum triglycerides				
No	reference		-	-
Yes	0.87 [0.77, 0.97]	**0.016**	-	-
Low serum HDL-cholesterol				
No	reference		reference	
Yes	1.08 [0.98, 1.20]	0.116	1.06 [1.00, 1.11]	**0.049**
Metabolic syndrome				
No	-	-	reference	
Yes	-	-	1.02 [0.96, 1.09]	0.544
Anemia				
No	reference		reference	
Yes	1.14 [1.04, 1.26]	**0.007**	1.02 [0.97, 1.08]	0.441
Iron deficiency				
No	reference		reference	
Yes	1.00 [0.91, 1.10]	0.990	1.01 [0.96, 1.06]	0.743
Vitamin A deficiency				
No	-	-	reference	
Yes	-	-	1.15 [0.98, 1.34]	0.078
Folate deficiency				
No	-	-	reference	
Yes	-	-	1.10 [1.04, 1.17]	**0.001**
Vitamin B12 deficiency				
No	reference		reference	
Yes	1.29 [1.19, 1.41]	**<0.001**	1.11 [1.06, 1.16]	**<0.001**

^a^ aPR: adjusted prevalence ratio. ^b^ Significant *p*-values, highlighted in bold, indicate statistical differences between the subgroup and the reference group.

## Data Availability

The data presented in this study are available on request from the corresponding author due to political reasons.

## Supplementary Materials

The following supporting information can be downloaded at https://www.mdpi.com/article/10.3390/nu17050804/s1. Table S1: Bivariate analysis of known risk factors for vitamin D deficiency and hypovitaminosis D. Table S2: Bivariate analysis of demographic and behavioral risk factors for vitamin D deficiency and hypovitaminosis D. Table S3: Bivariate analysis of metabolic and nutritional risk factors for vitamin D deficiency and hypovitaminosis D.

## Abbreviations

The following abbreviations are used in this manuscript:

25(OH)D	25-hydroxyvitamin D
AGP	α1-acid glycoprotein
aPR	adjusted prevalence ratio
AUB	American University of Beirut
AUBMC	American University of Beirut Medical Center
BMI	body mass index
CBC	complete blood count
CI	confidence interval
CRP	C-reactive protein
EA	enumeration area
EDTA	ethylenediaminetetraacetic acid
ELISA	enzyme-linked immunosorbent assay
FANTA	Food and Nutrition Technical Assistance
HbA1c	glycated hemoglobin
HDL	high-density lipoprotein
HFIAS	Household Food Insecurity Access Scale
IOM	Institute of Medicine
IS	informal settlement
kg	kilogram
L	liter
m	meter
LIMA	Lebanon Integrated Micronutrient, Anthropometry, and Child Development Survey
mL	milliliter
ng	nanogram
nmol	nanomole
RBP	retinol-binding protein
SF	serum ferritin
SGA	small children for gestational age
SST	serum-separating tubes
sTfR	soluble transferrin receptor
