# Supplementary material for: The Sunshine Paradox: Unraveling Risk Factors for Low Vitamin D Status Among Non-Pregnant Women in Lebanon
Source: Nutrients. 2025 Feb 26;17(5):804. doi: 10.3390/nu17050804 (PMC11901458; doi:10.3390/nu17050804)
Supplement: Supplementary file 1 [file nutrients-17-00804-s001.zip › nutrients-3479859-supplementary.pdf]

Table S1. Bivariate analysis of known risk factors for vitamin D deficiency and hypovitaminosis D.

Variables		Serum vitamin D (nmol/L)	Vitamin D deficiency		Hypovitaminosis D	
**Known risk factors**	**N**	**Median [95% CI]**	**% [95% CI]**	**p-value [a]**	**% [95% CI]**	**p-value [a]**
**Skin color**				0.147		**0.004**
Very white/white	1051	39.8 [35.4,44.1]	34.9 [30.0,40.1]		63.6 [57.9,68.8]	
Olive	1529	36.5 [33.8,39.2]	38.8 [34.4,43.3]		71.4 [67.0,75.4]	
Dark/very dark	213	33.0 [27.2,38.8]	45.6 [34.9,56.7]		79.1 [69.9,86.0]	
**Wearing hijab**				**<0.001**		**<0.001**
No	874	49.5 [46.4,52.6]	13.2 [9.3,18.3]		51.3 [44.8,57.7]	
Yes	1920	27.5 [25.9,29.1]	55.5 [51.8,59.1]		81.9 [79.1,84.4]	
**Daily sun exposure**				**0.023**		0.198
<1 h	510	32.5 [28.1,36.9]	45.8 [38.1,53.7]		74.9 [66.3,82.0]	
1-2 h	473	39.8 [35.3,44.2]	32.5 [24.9,41.1]		65.5 [58.0,72.2]	
2-3 h	473	40.5 [35.6,45.4]	34.4 [27.4,42.2]		66.5 [58.3,73.9]	
>3 h	873	39.5 [35.7,43.3]	33.4 [27.7,39.6]		68.0 [62.4,73.0]	
**Use of sunscreen**				**<0.001**		**<0.001**
No	1764	33.0 [30.6,35.4]	44.2 [39.6,49.0]		75.7 [71.3,79.6]	
Yes	1030	46.3 [43.2,49.3]	25.9 [21.9,30.3]		56.8 [51.2,62.3]	
**Vitamin D supplementation**				**<0.001**		**<0.001**
No	2247	33.0 [30.9,35.1]	44.6 [40.6,48.7]		76.9 [73.3,80.1]	
Yes	534	58.8 [54.4,63.1]	10.6 [7.2,15.4]		36.6 [30.5,43.3]	
**Multivitamin supplementation**				**0.032**		**<0.001**
No	2530	36.5 [34.1,38.9]	38.7 [35.1,42.4]		70.9 [67.5,74.0]	
Yes	240	50.5 [42.2,58.8]	29.1 [21.3,38.3]		48.4 [37.7,59.3]	

[a] significant p-values <0.05 were highlighted in bold and p-values less than 0.2 were included in the multivariate analysis.

Table S2. Bivariate analysis of demographic and behavioral risk factors for vitamin D deficiency and hypovitaminosis D

Variables		Serum vitamin D (nmol/L)	Vitamin D deficiency		Hypovitaminosis D	
**Demographic characteristics**	**N**	**Median [95% CI]**	**% [95% CI]**	**p-value [a]**	**% [95% CI]**	**p-value [a]**
**Age group in years**				**0.001**		**<0.001**
15-19	456	30.0 [25.1,34.9]	49.9 [42.1,57.7]		81.5 [73.9,87.3]	
20-29	751	36.0 [32.9,39.1]	39.5 [33.6,45.6]		74.6 [69.4,79.3]	
30-39	753	39.8 [33.4,46.1]	37.2 [30.7,44.2]		66.3 [60.3,71.8]	
40-49	843	41.0 [37.8,44.2]	31.1 [26.4,36.1]		61.1 [55.3,66.6]	
**Nationality**				**<0.001**		**<0.001**
Lebanese	2002	43.3 [40.8,45.7]	27.8 [24.4,31.5]		61.8 [57.5,65.9]	
Syrian	666	22.8 [20.3,25.2]	65.3 [58.4,71.6]		89.5 [84.7,92.9]	
Palestinian	97	24.0 [15.1,32.9]	57.1 [36.0,75.9]		80.2 [65.4,89.6]	
**Wealth quintile**				**<0.001**		**<0.001**
Lowest	677	25.3 [21.6,28.9]	59.4 [52.5,66.0]		84.5 [77.3,89.7]	
Second	525	34.3 [28.6,39.9]	42.1 [32.1,52.8]		78.7 [70.4,85.2]	
Middle	560	40.8 [36.9,44.6]	32.2 [25.8,39.3]		64.5 [56.9,71.5]	
Fourth	526	38.8 [34.7,42.8]	34.7 [28.6,41.2]		66.4 [59.4,72.7]	
Highest	515	51.3 [47.9,54.6]	15.8 [11.6,21.1]		47.7 [41.6,53.8]	
**Educational level**				**<0.001**		**<0.001**

Basic secondary or less	1488	30.8 [28.0,33.5]	49.0 [43.9,54.1]		77.7 [73.1,81.8]	
Complete secondary or more	1306	45.5 [42.7,48.3]	24.0 [20.5,28.0]		58.5 [53.5,63.2]	
**Household food security access**				**<0.001**		**<0.001**
Severe	775	28.5 [25.8,31.2]	54.3 [48.3,60.2]		79.2 [72.8,84.3]	
Moderate	859	36.5 [32.0,41.0]	39.3 [32.5,46.5]		69.0 [63.3,74.2]	
Mild	272	40.8 [35.2,46.3]	35.7 [27.1,45.4]		67.8 [58.6,75.7]	
Secure	897	43.5 [41.2,45.8]	24.0 [20.0,28.6]		61.7 [56.3,66.8]	
**Behavioral characteristics**						
**Minimum dietary diversity**				**0.001**		0.630
No	1284	34.0 [30.7,37.3]	43.7 [38.5,49.0]		70.1 [64.1,75.5]	
Yes	1510	39.5 [36.6,42.4]	33.5 [29.5,37.7]		68.5 [64.3,72.3]	
**Tobacco smoking**				**0.040**		**0.033**
No	1846	36.0 [33.1,38.9]	39.9 [35.8,44.2]		71.4 [67.2,75.3]	
Yes	948	40.5 [35.8,45.2]	33.9 [29.0,39.1]		64.8 [59.1,70.1]	
**Currently breastfeeding**				0.148		0.066
No	2715	37.5 [34.8,40.2]	37.6 [34.0,41.3]		68.9 [65.2,72.3]	
Yes	88	31.0 [26.7,35.3]	47.7 [34.5,61.2]		80.7 [67.8,89.3]	

[a] significant p-values <0.05 were highlighted in bold and p-values less than 0.2 were included in the multivariate analysis.

Table S3. Bivariate analysis of metabolic and nutritional risk factors for vitamin D deficiency and hypovitaminosis D

Variables		Serum vitamin D (nmol/L)	Vitamin D deficiency		Hypovitaminosis D	
**Metabolic disturbances**	**N**	**Median [95% CI]**	**% [95% CI]**	**p-value [a]**	**% [95% CI]**	**p-value [a]**
**BMI classes**				0.378		**0.002**
Underweight	121	34.3 [28.8,39.7]	40.3 [25.3,57.4]		85.1 [72.4,92.5]	
Healthy weight	1076	39.0 [34.4,43.6]	36.6 [31.0,42.6]		64.8 [59.4,69.8]	
Overweight	792	38.5 [34.7,42.3]	35.6 [30.9,40.6]		67.1 [61.2,72.6]	
Obese	743	34.5 [30.7,38.3]	42.5 [36.4,48.8]		75.6 [69.7,80.7]	
**Elevated HbA1c**				0.410		0.338
No	2429	36.8 [33.8,39.7]	38.5 [34.8,42.4]		68.9 [65.0,72.6]	
Yes	335	38.3 [33.6,42.9]	34.1 [24.9,44.5]		73.1 [64.7,80.1]	
**Elevated serum triglycerides**				0.113		0.555
No	2204	36.3 [33.7,38.8]	39.2 [35.5,43.1]		69.7 [66.1,73.1]	
Yes	599	39.3 [34.3,44.2]	33.2 [26.8,40.2]		67.6 [60.3,74.2]	
**Low serum HDL-cholesterol**				**0.014**		**0.001**
No	1431	39.8 [36.2,43.3]	34.4 [30.3,38.9]		64.7 [60.0,69.2]	
Yes	1372	34.5 [32.0,37.0]	41.8 [37.0,46.6]		74.2 [69.8,78.2]	
**Elevated blood pressure**				0.738		0.679
No	2604	37.0 [34.2,39.8]	37.9 [34.5,41.4]		68.8 [65.3,72.1]	
Yes	135	38.3 [32.1,44.4]	35.4 [22.1,51.4]		71.3 [57.7,81.9]	
**Visceral/central obesity**				0.392		0.060
No	1275	38.8 [34.6,42.9]	36.6 [32.1,41.5]		66.5 [61.5,71.1]	
Yes	1463	36.0 [33.2,38.8]	39.2 [34.6,44.0]		71.6 [67.3,75.6]	
**Metabolic syndrome**				0.387		**0.004**
No	2043	38.5 [35.5,41.5]	37.2 [33.8,40.8]		66.7 [62.6,70.6]	
Yes	673	35.0 [31.2,38.8]	39.6 [32.9,46.8]		76.6 [70.5,81.7]	
**Inflammation**				0.417		0.467
No	1613	36.8 [33.5,40.0]	38.9 [34.9,43.1]		68.1 [63.4,72.5]	
Yes	1159	37.5 [33.8,41.2]	36.4 [31.3,41.8]		70.4 [65.4,74.9]	

**Nutritional status**						
**Anemia**				**0.011**		**0.039**
No	1883	38.8 [36.0,41.5]	35.0 [31.0,39.2]		67.1 [62.8,71.2]	
Yes	903	34.0 [30.9,37.1]	43.4 [37.8,49.2]		73.2 [68.2,77.7]	
**Iron deficiency**				0.102		**0.017**
No	1680	38.5 [34.9,42.1]	35.7 [31.3,40.4]		66.5 [62.4,70.5]	
Yes	1092	35.0 [32.2,37.8]	41.2 [36.2,46.4]		73.2 [68.0,77.8]	
**Iron deficiency anemia**				**0.022**		**0.028**
No	2147	38.5 [35.5,41.5]	35.8 [31.9,39.9]		67.3 [63.3,71.1]	
Yes	612	34.0 [30.6,37.4]	44.4 [37.9,51.1]		74.9 [68.5,80.4]	
**Vitamin A deficiency**				0.426		**0.001**
No	2736	37.5 [34.8,40.2]	37.7 [34.1,41.4]		68.7 [65.1,72.1]	
Yes	36	30.0 [19.4,40.6]	48.9 [23.8,74.6]		95.5 [81.6,99.0]	
**Folate deficiency**				0.404		0.131
No	2295	38.3 [35.3,41.2]	37.3 [33.5,41.2]		68.1 [64.1,71.8]	
Yes	508	34.3 [30.9,37.6]	40.8 [33.4,48.6]		74.7 [66.6,81.3]	
**Vitamin B12 deficiency**				**<0.001**		**<0.001**
No	2186	41.3 [38.1,44.4]	31.5 [28.1,35.0]		64.3 [60.2,68.3]	
Yes	617	25.5 [23.5,27.5]	62.3 [54.5,69.6]		87.9 [83.9,91.0]	
**Zinc deficiency**				0.538		0.426
No	349	34.5 [27.5,41.5]	43.2 [34.2,52.8]		68.7 [60.8,75.6]	
Yes	191	36.5 [28.8,44.2]	37.9 [25.8,51.8]		74.2 [62.8,83.0]	

[a] significant p-values <0.05 were highlighted in bold and p-values less than 0.2 were included in the multivariate analysis.
